# Digital Rectal Examination in Stockholm3 Biomarker-based Prostate Cancer Screening

**DOI:** 10.1016/j.euros.2022.08.006

**Published:** 2022-08-29

**Authors:** Joel Andersson, Thorgerdur Palsdottir, Anna Lantz, Markus Aly, Henrik Grönberg, Lars Egevad, Martin Eklund, Tobias Nordström

**Affiliations:** a Department of Medical Epidemiology and Biostatistics, Karolinska Institutet, Stockholm, Sweden; b Department of Clinical Sciences, Danderyd Hospital, Karolinska Institutet, Stockholm, Sweden; c Department of Surgery, Capio St. Göran’s Hospital, Stockholm, Sweden; d Department of Urology, Karolinska University Hospital, Stockholm, Sweden; e Department of Molecular Medicine and Surgery, Karolinska Institutet, Stockholm, Sweden; f Department of Oncology-Pathology, Karolinska Institute, Stockholm, Sweden

**Keywords:** Diagnosis, Digital rectal examination, Prostate cancer, Prostate-specific antigen, Stockholm3 model

## Abstract

**Background:**

Pathological digital rectal examination (DRE) is suggestive of prostate cancer but has low sensitivity and specificity. DRE is incorporated in many clinical risk calculators, but there is less evidence on how DRE performs in the setting of blood biomarkers and polygenic risk prediction models other than prostate-specific antigen (PSA) associated with prostate cancer. The Stockholm3 test combines a blood test and clinical variables including DRE.

**Objective:**

To assess the predictive performance of DRE for finding clinically significant prostate cancer in systematic biopsy and evaluate its added value to the multivariable diagnostic test Stockholm3.

**Design, setting, and participants:**

This population-based study in the screening by invitation setting included 5543 men aged 50–69 yr with PSA ≥3 ng/ml who were referred for systematic prostate biopsy between 2012 and 2015. The STHLM3 study is registered with ISRCTN.com as ISRCTN84445406.

**Outcome measurements and statistical analysis:**

Predictive performance was assessed via estimates of sensitivity and specificity and in logistic regression. Clinically significant cancer was defined as International Society of Urological Pathology grade group ≥2 (GG ≥2) cancer on systematic biopsy.

**Results and limitations:**

We found that 11% of men with PSA ≥3 ng/ml had a suspicious DRE. A suspicious DRE was associated with a 3.16-fold higher risk (95% confidence interval [CI] 2.83–3.52) of GG ≥2 cancer and greater length of cancer on biopsy. The risk of nonsignificant cancer was similar regardless of the DRE finding. The risk of GG ≥2 cancer was 46.2% (95% CI 42.2–50.3%) for men with a suspicious DRE versus 14.6% (95% CI 13.7–15.7%) for men with a negative DRE. The elevated risk of GG ≥2 cancer persisted after adjusting for the other Stockholm3 test parameters (odds ratio 2.88, 95% CI 2.32–3.57). For detection of GG ≥2 cancer among men with PSA ≥3 ng/ml, DRE had sensitivity of 27.8% (95% CI 25.1–30.7%) and specificity of 92.8% (95% CI 92.1–93.6%).

**Conclusions:**

In this screening-by-invitation setting we found that for men with PSA ≥3 ng/ml, a suspicious DRE indicates more than threefold higher risk of harboring significant prostate cancer. DRE as a variable adds significant precision to the Stockholm3 prediction model. Men with a suspicious DRE should be referred for further diagnostic workup, including biopsy.

**Patient summary:**

We investigated the ability of digital rectal examination to predict if a patient has clinically significant prostate cancer. We found that digital rectal examination provides valuable information and can help doctors in making an informed decision on whether to recommend prostate biopsy.

## Take Home Message

A man with a positive digital rectal examination (DRE) has more than three times higher risk of harboring clinically significant prostate cancer on biopsy than a man with a negative DRE. We support the use of DRE as a second-line test after primary biomarker-driven testing strategies.

## 1 Introduction

Prostate-specific antigen (PSA) and digital rectal examination (DRE) have been cornerstones in the workup for detection of prostate cancer since the early 1990s [1]. In the European Randomised Study of Screening for Prostate Cancer (ERSPC) and the Prostate, Lung, Colorectal and Ovarian Cancer Screening Trial (PLCO), the two largest prostate cancer screening trials, DRE and PSA were initially used as screening tests. European and US guidelines state that a suspicious DRE is associated with an elevated risk of cancer and is an indication for biopsy [2], [3]. Since PSA <3 ng/ml has a low positive predictive value (PPV) for detection of clinically significant prostate cancer among men with suspicious DRE, use of DRE as a “tool” in screening has mostly been recommended after an initial PSA screening test [4], [5], [6]. DRE is one of the predictors in most clinical risk calculators for prostate cancer, including the ERSPC risk calculator [7], the Prostate Biopsy Collaborative Group calculator [8], and 4K. However, the performance of DRE for prostate biopsy decisions in early detection settings that include the use of polygenic markers in prediction models has not been fully elucidated.

The Stockholm3 test is a multivariable biomarker and clinical predictive model that decreases the number of biopsies needed to find International Society of Urological Pathology grade group ≥2 (GG ≥2) prostate cancer by 32% when maintaining the same sensitivity as a PSA cutoff of 3 ng/ml [9]. Apart from two clinical parameters that require clinical workup (DRE and prostate volume), all the other parameters in the model can be collected via a web questionnaire and a blood test.

We therefore sought to analyze the value of performing DRE as part of prostate cancer screening using data from a contemporary screening-by-invitation cohort of men. We evaluated the association between DRE status and the risk of clinically significant prostate cancer on transrectal systematic prostate biopsy and how the predictive performance of the Stockholm 3 model without DRE and prostate volume compares to the full model.

## 2 Patients and methods

The data used in this study were collected prospectively between 2012 and 2015 in the population-based STHLM3 diagnostic study [9]. Randomly sampled men aged 50–69 yr in Stockholm County, Sweden were invited for prostate cancer testing. The Stockholm3 test is based on a prediction model that includes a combination of plasma protein biomarkers (PSA, free PSA, intact PSA, hK2, MSMB, MIC1), genetic polymorphisms (232 single-nucleotide polymorphisms), and clinical variables (age, family, history, previous prostate biopsy, DRE). The STHLM3 study used a paired screen-positive design in which the Stockholm3 test was analyzed for all participants with PSA ≥1 ng/ml. Each participant was then recommended prostate biopsy if he had PSA ≥3 ng/ml or a Stockholm3 test probability of GG ≥2 cancer above a fixed threshold.

For this study, we included the men with PSA ≥3 ng/ml who underwent biopsy and had data on DRE status available. The biopsy procedure followed a standardized protocol for systematic biopsies. The physician performing the biopsy performed a DRE before biopsy, blinded to the indication for biopsy referral. DRE was rated as T1–T4 as an indication of the clinical T stage in diagnosing cancer. In this study, palpable T2–T4 status was defined as suspicious DRE (DRE^+^) and T1 was defined as nonsuspicious DRE (DRE^−^).

Our main definition of clinically significant prostate cancer was GG ≥2 cancer on systematic transrectal ultrasound-guided prostate biopsy. We also analyzed outcomes for GG ≥3 and any-grade prostate cancer. All biopsies were reviewed by a single pathologist (L.E.).

The STHLM3 study is registered on ISRCTN.com (ISRCTN84445406) and was approved by the institutional ethics review board.

### 2.1 Statistical analysis

A two-sided *t* test and χ^2^ test were used to assess differences in baseline variables between the suspicious and nonsuspicious DRE groups. The main outcome was the sensitivity and specificity of DRE as a diagnostic test for GG ≥2 cancer on biopsy. In addition, the same analyses were conducted for all prostate cancers and GG ≥3 cancer. Logistic multivariable regression analysis was used to evaluate prediction of GG ≥2 cancer with DRE, prostate volume, and a modified Stockholm3 model including only blood test parameters, age, and previous negative prostate biopsy (dichotomized as yes vs no) as predictors. Exclusion of clinical data on prostate volume and DRE from the full Stockholm3 model made it possible to analyze prostate volume and DRE independently. We constructed receiver operating characteristic (ROC) curves and compared the area under the curve (AUC) using the DeLong method [10]. Tests were performed for interaction between DRE and PSA, age, and the Stockholm3 result separately. To predict the risk of all cancers, GG ≥2 cancer, and GG ≥3 cancer, we ran three multivariable logistic regression models with DRE status and PSA levels from 3 to 20 ng/ml as predictors. Standard errors were calculated using the delta method and gave us a first-order approximation of the true standard error.

Missing prostate volume data were imputed using the overall median volume. Multivariable analysis including Stockholm3 was conducted on a complete-case basis.

Sensitivity analysis was carried out for the multivariable model excluding missing values for prostate volume.

No adjustments or corrections where made for multiple comparisons; individual *p* values and confidence intervals (CIs) should be interpreted with caution.

To assess the performance of a model using DRE in a clinical setting, we normalized results to a population of 1000 men with PSA ≥3 ng/ml. We calculated the reductions in the number of men undergoing biopsy and number of significant cancer cases found when biopsying men with PSA of either >4 or 3–4 ng/ml and with suspicious DRE in comparison to using PSA >3 ng/ml or >4 ng/ml alone as the cutoff. Statistical analysis was performed using Stata v14.2 (Stata Corp., College Station, TX, USA).

## 3 Results

### 3.1 Population characteristics

**Fig. 1:**
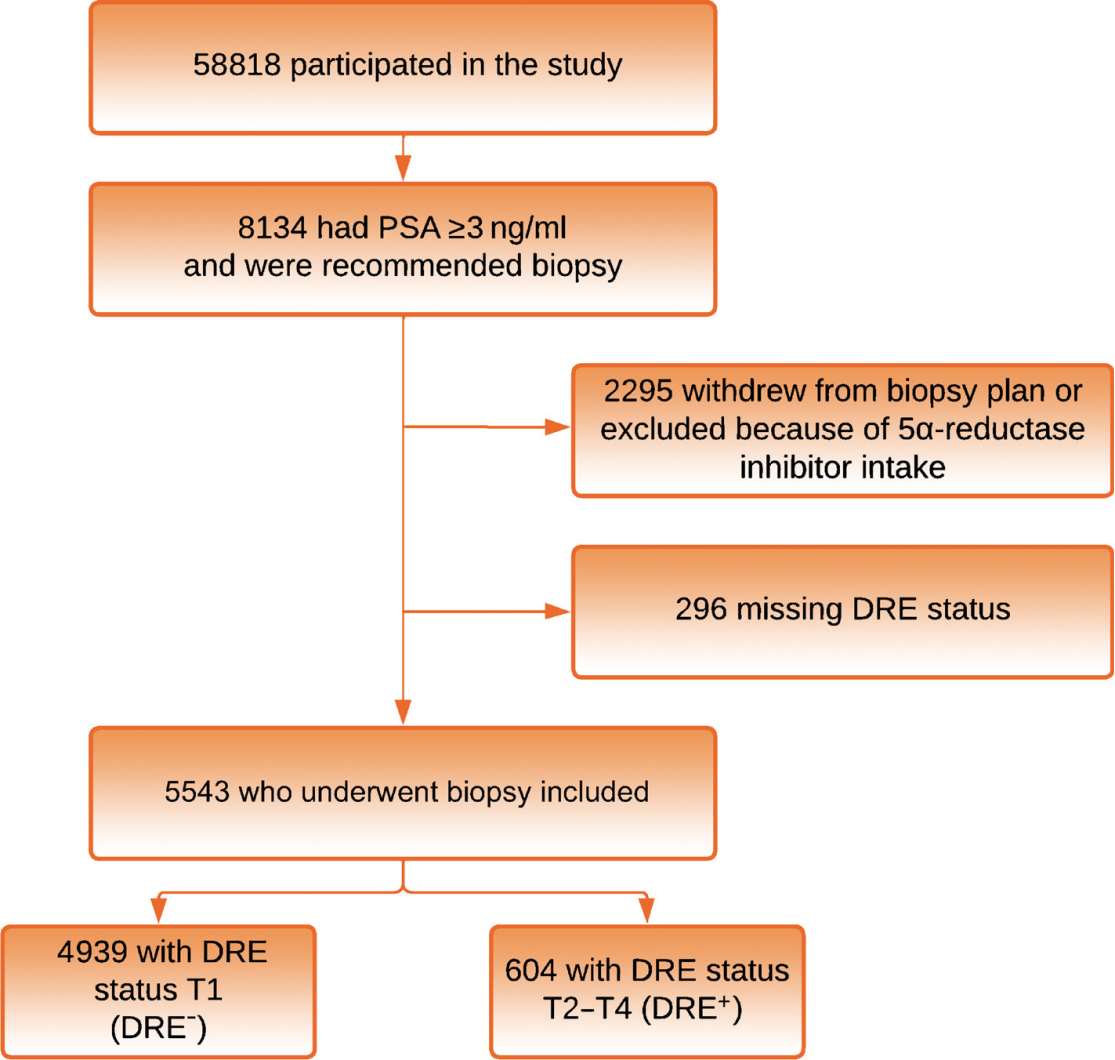
Study outline. PSA = prostate-specific antigen; DRE = digital rectal examination.

Figure 1 outlines the inclusion of men invited to participate in the STHLM3 screening-by-invitation study (*n* = 58 818). The participation rate was 41% and biopsy compliance was 72% (men biopsied/men recommended biopsy). In this cohort, 4939 out of 5543 (89.1%) men with PSA ≥3 ng/ml had nonsuspicious DRE and the remaining 604 (10.9%) had suspicious DRE. Data on prostate volume were missing for 218 subjects (4%) in the DRE^−^ group and 40 (7%) in the DRE^+^ group. The median prostate volume imputed was 43 cm^3^. A Stockholm3 test was missing for 272 subjects (5%). Clinical variables at baseline and biopsy outcomes are shown in Table 1. The DRE^+^ group had higher median PSA, smaller median prostate volume, and a lower rate of previous biopsy receipt in comparison to the DRE^−^ group (all *p* < 0.01; Table 1).

**Table 1:** Clinical characteristics and systematic prostate biopsy outcome in 5543 men with PSA **≥3** ng/ml

Variable	Negative DRE		Positive DRE		*p* value
Patients, *n* (%)	4939 (89.1)		604 (10.9)		
Median age, yr (IQR)	64.6 (59.7–67.5)		65.4 (60.8–67.8)		0.009
PSA (ng/ml)	4.2 (3.4–5.5)		4.8 (3.6–9)		<0.001
Median PV, cm^3^ (IQR)	43 (34–57)		40 (29.6–48)		<0.001
Previous biopsy, *n* (%)	379 (7.7)		27 (4.5)		0.004
Family history, *n* (%)	678 (13.7)		90 (14.9)		0.44
Cancer grading	*n* (%)	ACL, mm (95% CI)	*n* (%)	ACL, mm (95% CI)	
Benign	3187 (64.5)	–	220 (36.4)	–	
ISUP grade 1	1032 (20.9)	4.6 (4.2–5.0)	105 (17.4)	6.7 (5.2–8.3)	0.002
ISUP grade 2	476 (9.6)	14.6 (13.3–15.8)	118 (19.5)	25.5 (22.1–29.0)	<0.001
ISUP grade 3	135 (2.7)	18.6 (15.0–22.2)	69 (11.4)	38.8 (31.9–45.8)	<0.001
ISUP grade 4	52 (1.1)	16.1 (10.6–21.6)	37 (6.1)	39.6 (31.0–50.0)	<0.001
ISUP grade 5	57 (1.2)	34.5 (27.0–42.0)	55 (9.1)	67.1 (54.9–77.9)	<0.001
All cancer	1752 (35.5)		384 (63.6)		

ACL = average cancer length; DRE = digital rectal examination for suspicion of prostate cancer; IQR = interquartile range; ISUP = International Society of Urological Pathology; PSA = prostate-specific antigen; PV = prostate volume.

### 3.2 Diagnosis of significant cancer

The prevalence of GG ≥2 cancer was 18% (1002/5543) in the overall biopsied population. The proportion of men with GG 1 cancer was 20.9% (95% CI 19.8–22.1%) in the DRE^+^ group versus 17.4% (95% CI 14.4–20.6%) in the DRE^−^ group. A finding of higher-grade disease was more common in the DRE^+^ group, with a 3.16-fold higher risk (95% CI 2.83–3.52) of GG ≥2 cancer. Furthermore, among men with GG ≥2 cancer the average cancer length on biopsy was 38.9 mm (95% CI 35.1–42.7) in the DRE^+^ cohort versus 17.0 mm (95% CI 15.7–18.4) in the DRE^−^ cohort (Table 1).

### 3.3 Predictive performance

Among men with PSA ≥3 ng/ml, DRE had sensitivity of 28% for detection of GG ≥2 cancer. The specificity, PPV, and negative predictive value were 93%, 46%, and 85%, respectively. Sensitivity for any-grade cancer was 18%, with a PPV of 64% (Table 2).

**Table 2:** Sensitivity, specificity, PPV, and NPV for positive DRE status in detecting all cancers, GG **≥2** cancer, and GG **≥3** cancer among men with prostate-specific antigen **≥3** ng/ml

	All prostate cancer (prevalence 39%)	GG ≥2 cancer (prevalence 18%)	GG ≥3 cancer (prevalence 7.3%)
Sensitivity, % (95% CI)	17.9 (16.3–19.6)	27.8 (25.1–30.7)	39.8 (35.0–44.7)
Specificity, % (95% CI)	93.5 (92.7–94.3)	92.8 (92.1–93.6)	91.4 (90.6–92.1)
RR (95% CI)	1.79 (1.66–1.92)	3.16 (2.83–3.52)	5.40 (4.51–6.46)
PPV, % (95% CI)	63.6 (59.6–67.4)	46.2 (42.2–50.3)	26.7 (23.2–30.4)
NPV, % (95% CI)	64.4 (63.1–65.8)	85.4 (84.3–86.3)	95.1 (94.4–95.6)
Likelihood ratio for positive DRE	2.78 (2.37–3.25)	3.89 (3.37–4.5)	4.61 (3.97–5.35)

CI = confidence interval; DRE = digital rectal examination; GG = International Society of Urological Pathology grade group; NPV = negative predictive value; PPV = positive predictive value; RR = relative risk of finding specified cancer for positive compared to negative DRE status.

In the multivariable regression model including prostate volume and the modified Stockholm3 model without prostate volume and DRE status as predictors, the odds ratio for the risk of GG ≥2 cancer for DRE^+^ status was 2.88 (95% CI 2.32–3.57; Table 3). The increase in AUC was small but statistically significant when DRE was added as a predictor to a model already including both prostate volume and the information in the Stockholm3 test (0.785 vs 0.775; *p* = 0.001; Table 4).

**Table 3:** Univariate and multivariable logistic regression for predicting the risk of clinically significant prostate cancer on biopsy for 5839 men with prostate-specific antigen **≥3** ng/ml with DRE status, prostate volume, and the Stockholm3 result as independent variables

	Odds ratio (95% CI)	*p* value
Univariate		
Unsuspicious DRE	1.00 (reference)	
Suspicious DRE	5.01 (4.19–5.98)	<0.001
Multivariable		
Positive DRE	2.88 (2.32–3.57)	<0.001
Prostate volume (in ml)	0.98 (0.97–0.98)	<0.001
Stockholm3^a^ (% predicted risk)	1.05 (1.05–1.06)	<0.001

CI = confidence interval; DRE = digital rectal examination.

a The Stockholm3 model with genetic, biochemical, and some clinical data (previous biopsy and age) but not prostate volume or DRE status.

**Table 4:** AUC for the models with and without DRE status and prostate volume^a^

Model	AUC (95% CI)	*p* value^b^
Prostate-specific antigen	0.647 (0.627–0.668)	–
Prostate-specific antigen + prostate volume + DRE status	0.742 (0.726–0.760)	<0.001
Stockholm3^c^	0.752 (0.734–0.770)	<0.001
Stockholm3^c^ + prostate volume	0.773 (0.756–0.790)	<0.001
Stockholm3^c^ + prostate volume + DRE status	0.784 (0.767–0.801)	0.001

AUC = area under the receiver operating characteristic curve; DRE = digital rectal examination.

a Models with prostate-specific antigen and prostate-specific antigen + prostate volume + DRE are included for reference.

b Test of significance for the null hypothesis: H_0_: AUC of model = AUC of model in the row above.

c The Stockholm3 model with genetic, biochemical, and some clinical data (previous biopsy and age) but not prostate volume or DRE status.

**Fig. 2:**
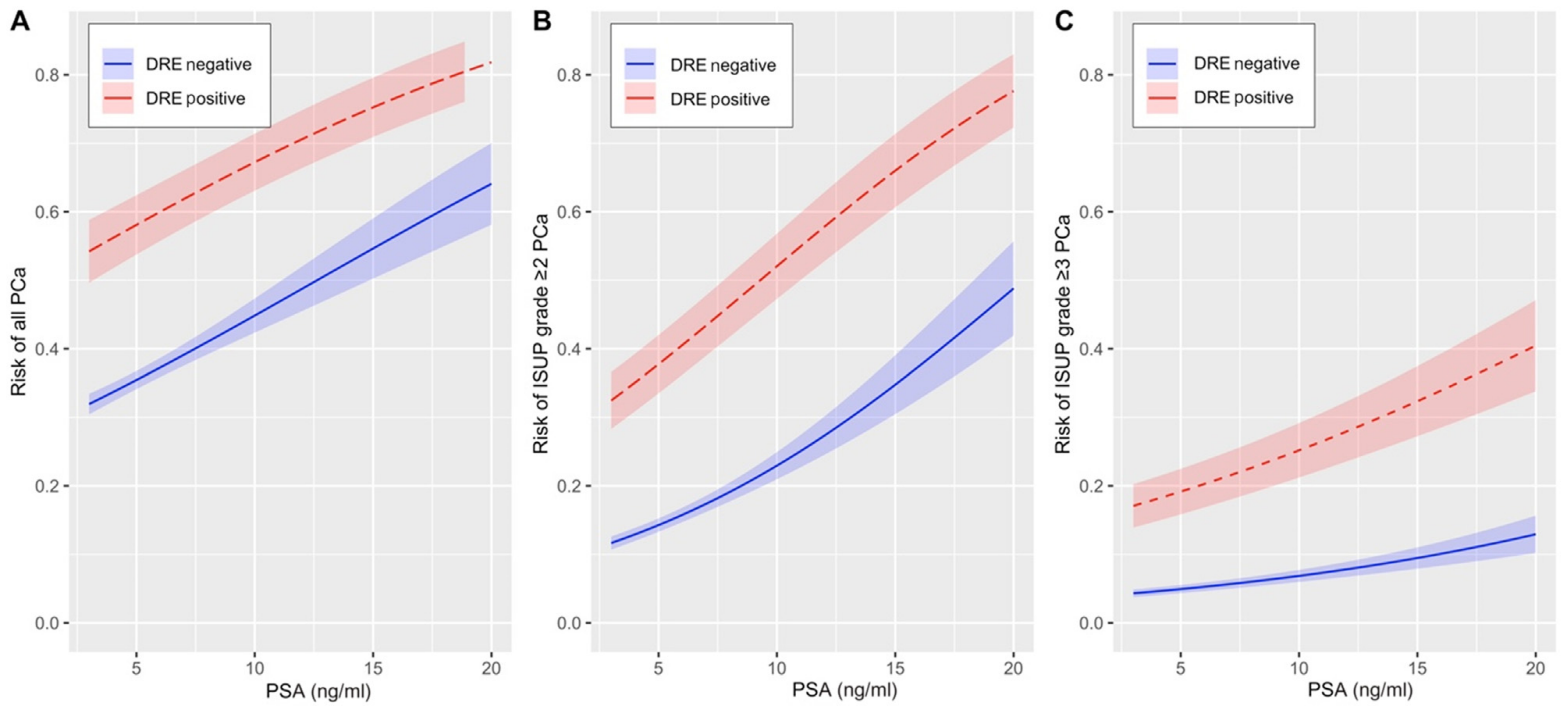
Predicted risk of prostate cancer as a function of PSA and DRE for (A) all cancer, (B) ISUP grade ≥2 cancer, and (C) ISUP grade ≥3 cancer. The bold lines show the predicted means and shaded areas denote the 95% confidence intervals. Multivariable logistic regressions were used to model the predicted risk with DRE and PSA as predictive variables. The graphs are limited to PSA between 3 and 20 ng/ml. PSA = prostate-specific antigen; DRE = digital rectal examination; ISUP = International Society of Urological Pathology; PCa = prostate cancer.

Figure 2 shows the probability of finding any-grade cancer, GG ≥2 cancer, and GG ≥3 cancer by DRE status and PSA level. For the PSA range 3–20 ng/ml, the absolute risk difference for men with DRE^+^ status was 22–33% for GG ≥2 cancer (Fig. 2B) and 13–32% for GG ≥3 cancer (Fig. 2C). A similar association was seen on stratification by PSA density (<0.15 vs ≥0.15 ng/ml/cm^3^; Supplementary Fig. 1).

Analysis revealed no significant interaction between DRE and age, Stockholm3 result, or PSA for GG ≥2 cancer as the outcome (Supplementary Table 2).

In the sensitivity analysis in which cases with missing data for prostate volume were excluded from the multivariable regression, the results did not change materially (Supplementary Table 3).

### 3.4 Proposed model and head-to-head comparison

Supplementary Table 1 shows predictions normalized to a population of 1000 men with PSA ≥3 ng/ml considered for further workup in a prostate cancer screening setting. We found that using a PSA threshold of either ≥4 ng/ml or 3–4 ng/ml and DRE+ status would lead to 40% fewer biopsies, but at the cost of 24% fewer GG ≥2 cancer cases identified. Using these data, 49 men with PSA of 3–4 ng/ml would need to undergo DRE, resulting in biopsy for four men that would detect 1.9 cancers and one clinically significant cancer.

## 4 Discussion

Our analysis of men from a large population-based screening study shows that DRE has sensitivity of 28% and specificity of 93% for predicting GG ≥2 cancer among men with PSA ≥3 ng/ml. These results are congruent with previous findings [11], [12]. With specificity of 93% and a PPV of 46%, a suspicious DRE can aid in identifying men at higher risk of clinically significant cancer in this group.

We found that approximately one in ten men with PSA ≥3 ng/ml had a suspicious DRE and the proportion increased with higher PSA level. For men with a suspicious DRE the risk of having ISUP ≥2 cancer was nearly 50%, which is more than three times higher than the risk for men with a nonsuspicious DRE. In comparison to findings from the ERSPC (35%) [7] and PLCO (15.6%) [13] trials, we found that DRE had a higher PPV for GG ≥2 cancer. The difference is mostly explained by differences in population selection, mainly because we used a PSA cutoff of ≥3 ng/ml. In the DRE^+^ group the proportion of men with a previous negative biopsy was 42% less than in the DRE^−^ group. This suggests that at a population level, the extent of prescreening for cancer before possible inclusion in this study could have been greater for the DRE^+^ group. This could possibly explain the lower DRE^+^ rate in our study than in previous screening trials. For instance, more than 20% of the men in the ERSPC Rotterdam cohort had a suspicious DRE in each of the different screening rounds [11].

Our results show that a DRE suspicious for cancer is strongly correlated with higher-grade cancer and greater cancer length on biopsy, confirming previous data on a larger volume of cancer in the prostate for men with a suspicious DRE [2]. The PCPT and PLCO trials showed an interaction between DRE status and PSA level for the risk of GG ≥2 cancer [4], [14], but our data do not support such an interaction.

Our results support the recommendation that patients with a suspicious DRE should be investigated further for prostate cancer. Furthermore, the association with higher-grade cancer, greater cancer length on biopsy, and by definition a minimum of T2 stage would suggest higher clinically significance among DRE^+^ GG ≥2 cancers. The increase in AUC was statistically significant when DRE and prostate volume were added as predictors to the simplified Stockholm3 model using only blood test and questionnaire parameters (0.785 vs 0.752; *p* = 0.001). The small increase reflects the fact that many men would need the extra screening steps of DRE and measurement of prostate volume to find one significant cancer regardless of the probability threshold chosen as a cutoff for biopsy.

There are some practical concerns for DRE; because of its low sensitivity, DRE cannot be used as a “rule out” test, with an expected high false-negative rate. More than half of patients undergoing DRE experience non-negligible discomfort [15]. According to the data in Table 4, we can assume a relatively high number needed to screen among men with a lower risk threshold according to PSA and other parameters than in our population, such as PSA <3.0 ng/ml. We deliberately chose to exclude men in the STHLM3 study with PSA of 1–2.99 ng/ml because of the selection bias this would have introduced, since these men were biopsied on the basis of elevated risk according to the Stockholm3 model.

Optimal incorporation of DRE in screening for prostate cancer in relation to multiparametric magnetic resonance imaging (mpMRI) and guided biopsy needs further study. Morote et al [16] showed that use of mpMRI did not decrease the number of men needing biopsy in the subgroup with PSA ≥10 ng/ml and DRE^+^ in a study of 768 men scheduled for prostate biopsy. Use of DRE as a second-line test to provide clinical direction regarding patients who have had negative mpMRI and/or prostate biopsies is beyond the scope of this study, but there is recent evidence that mpMRI is more beneficial for DRE^−^ than for DRE^+^ men in finding clinically significant prostate cancer [17].

The main strength of our study is the large population-based invitational setting. DRE was performed by urology specialists, blinded to the PSA and Stockholm3 test results, and there was a clear definition of DRE^+^ status in terms of T stage. Biopsy compliance for patients for whom biopsy was recommended was moderate at 71% (Fig. 1). A urologist performed systematic biopsies according to a predefined scheme. The pathology analysis was performed by an expert uropathologist who was blinded to T-stage assessment. This makes the risk of selection bias and misclassification bias low. The lack of mpMRI information in our study is the main limitation. We cannot say what independent predictive value DRE has in a risk model that includes mpMRI information. Our definition of a true positive as GG ≥2 cancer in one round of 10–12 systematic peripheral-zone transrectal biopsies is another limitation. The PROMIS study showed that systematic transrectal biopsy diagnosed only 48% of clinically significant prostate cancers in comparison to template prostate mapping biopsy [18]. Up to 30% of cancers are in the anterior transitional zone and these are less likely to be palpable [19]. Our biopsy scheme is not specifically aimed at the anterior zone, and this is a possible area of bias. In contemporary recommendations for early detection, mpMRI targeted biopsy is strongly recommended and has been established as a standard of care in settings where mpMRI is available [3], [20]. From this study we cannot say how DRE performs with regard to any long-term oncological results such as survival or upgrading at radical prostatectomy, or how DRE performs among men with PSA <3 ng/ml.

## 5 Conclusions

DRE suspicious for cancer is highly associated with elevated risk of significant prostate cancer. Thus, prostate biopsy should be recommended for men with abnormal DRE findings. DRE and prostate volume add significant predictive value to the performance of the Stockholm3 model.

  ***Author contributions***: Joel Andersson had full access to all the data in the study and takes responsibility for the integrity of the data and the accuracy of the data analysis.

*Study concept and design*: Grönberg, Eklund.

*Acquisition of data*: Egevad, Aly, Nordström.

*Analysis and interpretation of data*: Eklund, Andersson, Palsdottir.

*Drafting of the manuscript*: Andersson.

*Critical revision of the manuscript for important intellectual content*: Nordström, Eklund, Palsdottir, Aly, Lantz.

*Statistical analysis*: Andersson, Palsdottir, Eklund.

*Obtaining funding*: Grönberg, Eklund, Nordström.

*Administrative, technical, or material support*: Grönberg.

*Supervision*: Nordström.

*Other*: None.

  ***Financial disclosures:*** Joel Andersson certifies that all conflicts of interest, including specific financial interests and relationships and affiliations relevant to the subject matter or materials discussed in the manuscript (eg, employment/affiliation, grants or funding, consultancies, honoraria, stock ownership or options, expert testimony, royalties, or patents filed, received, or pending), are the following: Henrik Grönberg has five patents pending that are related to prostate cancer diagnostics, has patent applications licensed to A3P, and might receive royalties from sales related to these patents. Martin Eklund is named on four of these five patent applications. The remaining authors have nothing to disclose.

  ***Funding/Support and role of the sponsor*:** The main funder of the STHLM3 study was Stockholm County Council (Stockholms Läns Landsting), the main provider of health care in Stockholm. Auxiliary funding for pilots and infrastructure was provided by the Swedish Cancer Society (Cancerfonden), Restaurants Against Cancer (RAC), the Swedish Research Council (Vetenskapsrådet), Odd Fellow in Västerås, the Swedish Research Council for Health Working Life and Welfare (FORTE), and the Swedish e-Science Research Center. The STHLM3 study was a part of the Linnaeus Center CRISP “Predication and prevention of breast and prostate cancer” project funded by the Swedish Research Council. Joel Andersson was funded by Hagstrandska Minnesfonden. The sponsors had no direct role in the study.
