# Supplementary material for: Digital Rectal Examination in Stockholm3 Biomarker-based Prostate Cancer Screening
Source: Eur Urol Open Sci. 2022 Aug 29;44:69–75. doi: 10.1016/j.euros.2022.08.006 (PMC9520496; doi:10.1016/j.euros.2022.08.006)

# **Supplementary material**

Supplementary Table 1: Comparison of models for prompting biopsy in terms of reduced number of biopsies and missed cancers compared to a strategy where all men with prosatate specific antigen (PSA) ≥3 ng/ml undergoes biopsy. DRE+ = digital rectal exam suspicious for cancer.

|  | Biopsies | | All prostate cancer | | ISUP GG ≥2 cancer | | ISUP GG ≥3 cancer | |
| --- | --- | --- | --- | --- | --- | --- | --- | --- |
| Biopsy protocol | Performed  N (%) | Not performed N (%) | Detected  N (%) | Missed  N (%) | Detected N (%) | Missed N (%) | Detected N (%) | Missed N (%) |
| Biopsy all PSA ≥3 ng/ml | 1000 (100%) | 0 (0%) | 386 (100%) | ref | 181 (100%) | ref | 73 (100%) | Ref |
| Biopsy if PSA ≥4 or  3-3.99 and DRE+ | 607 (61%) | 393 (39%) | 259 (67%) | 127 (33%) | 138 (76%) | 43 (24%) | 60 (82%) | 13 (18%) |
| Biopsy if PSA ≥4 ng/ml | 569 (57%) | 431 (43%) | 242 (63%) | 144 (37%) | 128 (71%) | 53 (29%) | 55 (76%) | 18 (24%) |
| ref = reference protocol | | | | | | |  | |

Supplementary Table 2: Multivariable logistic regression on risk of presence of clinically significant prostate cancer on biopsy in 5,839 men with PSA≥3 ng/ml and area under the receiver-operating curve with interaction terms.

| Interaction between DRE and Age | Odds Ratio | p-value | [95% Confidence Interval] |
| --- | --- | --- | --- |
| DRE (+) | 8.16 | 0.08 | (0.78 - 85.5) |
| Age (years) | 1.02 | 0.02 | (1.003 - 1.035) |
| DRE * Age | 0.99 | 0.68 | (0.96 - 1.03) |
| Interaction between DRE and Prostatevolume |  |  |  |
| DRE (+) | 7.79 | <0.001 | (4.56 – 13.31) |
| Prostatevolume (ml) | 0.97 | <0.001 | (0.97 – 0.98) |
| DRE * Prostatevolume | 0.99 | 0.038 | (0.97 – 1.00) |
| Interaction between DRE and STHLM3 |  |  |  |
| DRE (+) | 3.15 | <0.001 | (2.21 - 4.49) |
| STHLM3 (% predicted risk) | 1.056 | <0.001 | (1.050 - 1.062) |
| DRE * STHLM3 | 0.999 | 0.93 | (0.987 – 1.012) |
| Interaction between DRE and PSA |  |  |  |
| DRE (+) | 3.99 | <0.001 | (2.88 – 5.53) |
| PSA (ng/ml) | 1.121 | <0.001 | (1.097 – 1.146) |
| DRE * PSA | 0.995 | 0.83 | (0.955 – 1.038) |
| * Interaction term | | | |

Table S3: Sensitivity analysis - Multivariable logistic regression on risk of presence of clinically significant prostate cancer on biopsy in 5,028 men (excluding 258 men with missing prostate volume data) with PSA≥3 ng/ml and area under the receiver-operating curve for models with or without DRE.

|  | Odds Ratio | p-value | [95% Confidence Interval] |
| --- | --- | --- | --- |
| DRE (+) | 2.81 | <0.001 | (2.25 - 3.51) |
|  | ROC AUC | p-value |  |
| PSA | 0.644 | reference | (0.623 - 0.665) |
| STHLM3* | 0.751 | <0.001^†^ | (0.732 - 0.769) |
| STHLM3* + Prostate volume | 0.772 | <0.001^†^ | (0.755 - 0.790) |
| STHLM3* + Prostate volume + DRE | 0.783 | 0.001^†^ | (0.766 - 0.800) |
| * The STHLM3 model with genetical, biochemical and some clinical data (previous biopsy and age) but not prostate volume or DRE status.  † Test of significance (H_0_: AUC of model = AUC of model in row above) | | | |

Figure S1: Risk-prediction of ISUP grade ≥2 prostate cancer as a function of prostate-specific antigen (PSA), DRE and PSA Density (<.15/ ≥.15 ng/ml/cc) using multivariable logistic regression. Bold and dashed line shows the predicted means and shaded area shows 95% confidence interval. Graph limited to PSA between 3 and 20 ng/ml.


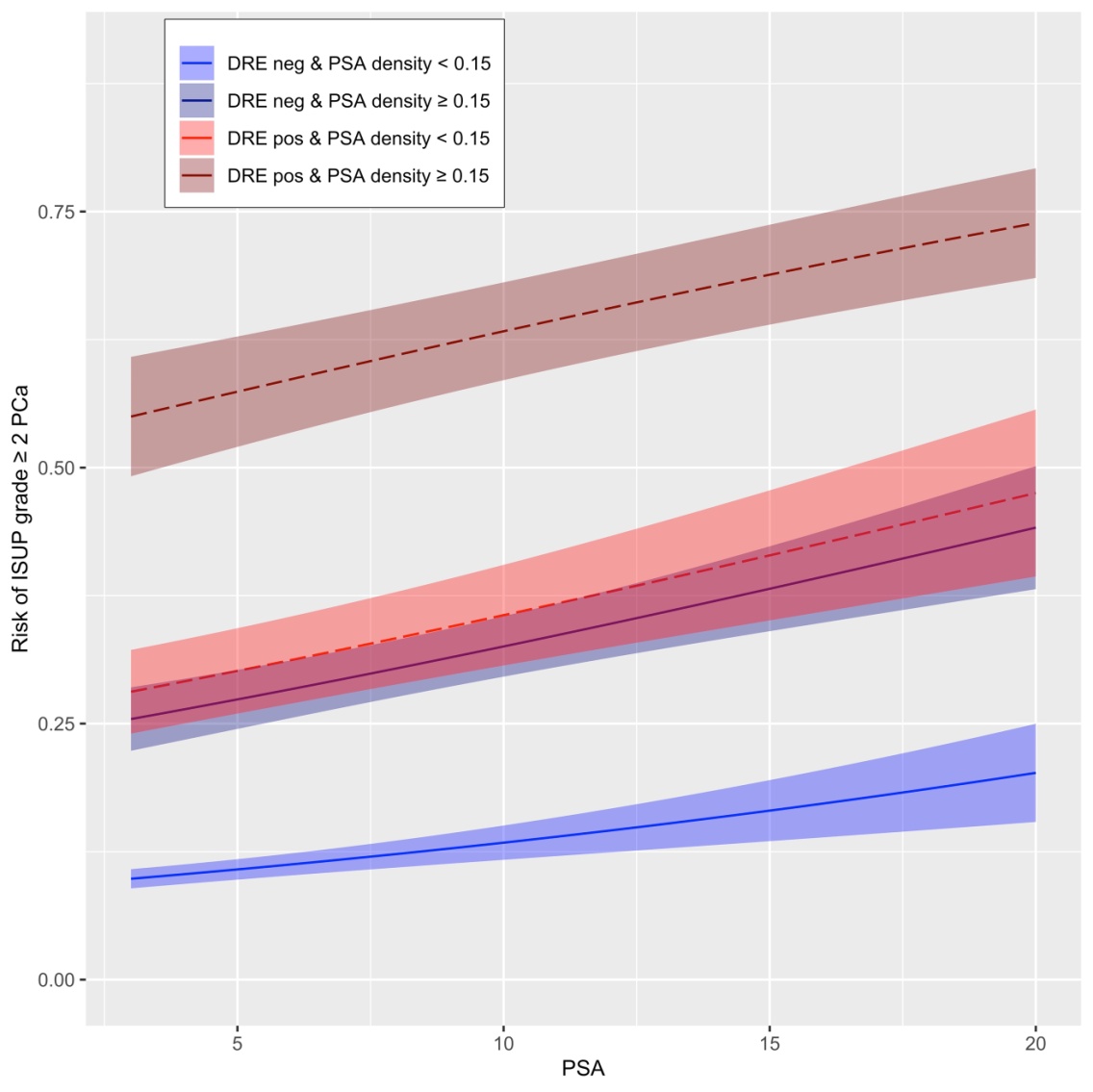

Supplement: Supplementary data 1 [file mmc1.docx]
